# Supplementary material for: Content-rich biological network constructed by mining PubMed abstracts
Source: BMC Bioinformatics. 2004 Oct 8;5:147. doi: 10.1186/1471-2105-5-147 (PMC528731; doi:10.1186/1471-2105-5-147)
Supplement: Additional File 2 — The original results of the above study (non-essential files are deleted to keep the file size under the limit set by BMC bioinformatics). [file 1471-2105-5-147-S2.bz2 › chilibotAdditionalFile2/dip10/459ID10488147E10477/html/CX3CL1_CX3CR1.html]

 


 **CX3CL1** and **CX3CR1** 
  
Found 71 abstracts in PubMed, retrieved 10.  
 

 What does Google say? 
 PDF only 
| .edu only 

---

**Interactive relationship** (e.g. stimulation, inhibition, etc)

**Neutral relationship**- In contrast to CD16 monocytes, CD16 monocytes expressed high  **CX3CR1**  and CXCR4 but low CCR2 and CD62L levels and underwent efficient transendo thelial migration in response to fractalkine  [ **CX3CL1** ]  FKN.  Ref: 12810688 J Exp Med, 2003

**Non-interactive relationship** (e.g. studied together, co-existance, homology, etc.)

- Consistent with this, intravenous injection of a single dose of recombinant IL 15 into C57BL 6 mice decreased steady state  **CX3CR1**  levels 24 hours after injection in freshly isolated PBMCs, splenocytes and bone marrow cells, and treatment of mouse PBMCs with IL 15 in vitro inhibited  **CX3CL1**  induced chemotaxis.  Ref: 12881312 BloodBlood, 2003
- Transient transfections with dnIkappaB alpha, dnIkappaB beta, dnIKK gamma, kdNIK, and kdIKK beta or pretreatment with wortmannin, Akt inhibitor, PDTC, or MG 132 significantly attenuated TNF alpha induced fractalkine  [ **CX3CL1** ]  and  **CX3CR1**  expression.  Ref: 12729461 Biochem J< MedlineTA>Biochem J, 2003
- Ad35K infected cell culture medium, containing recombinant 35K, potently reduced migration of CCR5 transfected 293 cells by 95% in response to the CC chemokine RANTES, but had no effect on cells transfected with the  **CX3CR1**  fractalkine  [ **CX3CL1** ]  receptor.  Ref: 12777060 Immunobiology, 2003
- Fractalkine  [ **CX3CL1** ]   **CX3CL1**  is a chemokine which is expressed predominantly in the central nervous system, being localized on neurons, while its receptor,  **CX3CR1** , is found on microglial cells.  Ref: 12725801 Methods, 2003
- Interactions between fractalkine  [ **CX3CL1** ]  and  **CX3CR1**  can mediate not only chemotaxis but also cell adhesion in the absence of substrates for other adhesion molecules.  Ref: 12806431 Drug News Perspect, 2001
- Both fractalkine  [ **CX3CL1** ]  and  **CX3CR1**  were expressed constitutively in neurons, microglia, and astrocytes.  Ref: 12850572 Brain Res, 2003
- Fractalkine  [ **CX3CL1** ]  receptor  **CX3CR1**  is expressed on natural killer NK cells, monocytes and some portions of CD8 T cells.  Ref: 12806431 Drug News Perspect, 2001
- OBJECTIVE To investigate the expression of fractalkine  [ **CX3CL1** ]  FKN and its receptor  **CX3CR1**  in cardiac allografts and the effect of Cyclosporin A CsA.  Ref: 12783679 Zhonghua Wai Ke Za Zhi, 2003
- OBJECTIVESWe have recently reported that the polymorphism of the fractalkine  [ **CX3CL1** ]  receptor,  **CX3CR1** , provides a new marker for prognosis in HIV disease.  Ref: 12799549 AIDS, 2003
- Pretreatment with pertussis toxin or anti  **CX3CR1**  neutralizing antibodies attenuated TNF alpha induced fractalkine  [ **CX3CL1** ]  induction indicating that fractalkine  [ **CX3CL1** ]  autoregulation plays a role in TNF alpha induced sustained fractalkine  [ **CX3CL1** ]  expression.  Ref: 12729461 Biochem J< MedlineTA>Biochem J, 2003
- TNF alpha activated NF gammaB and induced fractalkine  [ **CX3CL1** ]  and  **CX3CR1**  expression in a time dependent manner in rat aortic smooth muscle cells.  Ref: 12729461 Biochem J< MedlineTA>Biochem J, 2003
- In the present study, we examined the production of fractalkine  [ **CX3CL1** ]  and the expression of its receptor,  **CX3CR1**  in murine glial and neuronal cell in vitro, and investigated its neuroprotective functions.  Ref: 12850572 Brain Res, 2003
